# Supplementary material for: QTL Mapping for Leaf Rust Resistance in a Common Wheat Recombinant Inbred Line Population of Doumai/Shi4185
Source: Plants (Basel). 2025 Oct 9;14(19):3113. doi: 10.3390/plants14193113 (PMC12526481; doi:10.3390/plants14193113)
Supplement: Supplementary file 1 [file plants-14-03113-s001.zip › plants-3857649-supplementary.pdf]

Table S1. The diverse panel used for KASP markers validation

Name	*KASP-LR-2AL*	*KASP-LR-7BL2*	Mean MDS (%)
Doumai	AA	TT	60
Han6172	GG	TT	70
Hengguan35	AA	TT	65
Huaimai20	GG	TT	60
Jimai22	AA	TT	60
Jimai23	AG	TT	65
Jinmai45	AA	TT	60
Jinmai61	AA	TT	70
Jing411	AA	TT	65
Jing9428	AA	TT	60
Jingdong17	AA	TT	60
Jingdong22	AA	TT	55
Jingdong8	AA	TT	65
Keheng6654	GG	TT	65
Lankao24	GG	TT	55
Liangxing66	AG	TT	70
Liangxing99	AG	TT	60
Linmai2	GG	TT	55
Linmai4	GG	TT	55
Lumai11	GG	TT	60
Lumai14	AA	TT	20
Lumai15	GG	TT	30
Lumai23	GG	TT	55
Lumai6	GG	TT	55
Lumai9	AA	TT	65
Ningdong10	AA	TT	55
Ningdong11	AA	TT	65
Nongda139	GG	TT	60
Nongda211	AA	TT	35
Nongda212	AA	TT	40
Shan229	AA	TT	40
Shijiazhuang8	AA	TT	65
Taishan1	GG	TT	60
Wan23094	GG	TT	45
Wanmai38	GG	TT	40
Wennong14	AG	TT	55
Xinong291	AA	TT	60
Xinong88	AA	TT	45
Xinong979	GG	TT	45
Xiaoyan22	GG	TT	50
Xinmai26	GG	TT	50
Xinmai37	AA	TT	65
Yumai13	GG	TT	35
Yumai21	GG	TT	55
Yumai34	AA	TT	50
Yumai47	GG	TT	45
Yumai7	AG	TT	60

Zhengmai366	GG	TT	50
Zhengyin1	GG	TT	50
Zhengzhou3	GG	TT	65
Zhong892	GG	TT	75
Zhongmai175	AA	TT	40
Zhongmai415	AA	TT	65
Zhongmai871	AA	TT	55
Zhongyou206	AA	TT	60
Zhongyou335	AA	TT	50
Zhongyou9507	AA	TT	65
Zhoumai12	AA	TT	55
Zhoumai13	GG	TT	10
Zhoumai16	GG	TT	35
Zhoumai25	AA	TT	40
Zhoumai28	GG	TT	45
Zimai12	GG	TT	10
Zixuan2	AG	TT	65
Lumai8	GG	TT	75
Sunong6	GG	TT	60
Xiaoyan81	GG	TT	75
Zhongyu9	GG	TT	65
Shannong20	AA	CC	20
Shan354	GG	CC	10
Shan512	GG	CC	8
Xinong2000-7	GG	CC	30
Xinmai9408	GG	CC	10
Zhoumai18	GG	CC	20
Abu	GG	CC	45
Aifeng3	GG	CC	45
Aikang58	GG	CC	60
Beijing0045	AA	CC	60
Beijing841	GG	CC	55
Fengkang2	AA	CC	60
Gaoyou503	GG	CC	25
Gaocheng8901	GG	CC	55
Hengguan33	AA	CC	60
Huapei5	AA	CC	50
Huaimai21	GG	CC	8
Jimai19	GG	CC	65
Jimai20	GG	CC	30
Jimai21	GG	CC	70
Jinan13	GG	CC	45
Jinan17	GG	CC	50
Jining16	GG	CC	60
Jishi02-1	GG	CC	50
Jinhe9123	GG	CC	60
Jinmai67	GG	CC	40
Jingshuang16	AA	CC	55
Lankao2	GG	CC	55

Lankao906	GG	CC	50
Linhan2	GG	CC	70
Linkang12	GG	CC	8
Lumai21	GG	CC	40
Lumai5	GG	CC	80
Luyuan502	AA	CC	50
Lunxuan987	GG	CC	40
Luomai21	GG	CC	65
Neixiang188	GG	CC	65
Qinnong142	GG	CC	40
Qinnong731	GG	CC	20
Shan253	GG	CC	45
Shanmai94	GG	CC	45
Shannong78-59	GG	CC	8
Shannong981	GG	CC	45
Shanyou225	GG	CC	40
Shi4185	GG	CC	45
Shijiazhuang15	AA	CC	65
Shixin733	AG	CC	45
Shixin828	GG	CC	45
Shiyou17	GG	CC	50
Taishan5	GG	CC	10
Wanmai29	GG	CC	20
Wanmai33	GG	CC	50
Wanmai50	GG	CC	55
Wanmai52	GG	CC	65
Wanmai53	GG	CC	55
Wennong5	GG	CC	65
Xinong1376	GG	CC	5
Xiaoyan54	GG	CC	20
Xiaoyan6	GG	CC	40
Xinmai19	GG	CC	10
Yannong15	GG	CC	60
Yannong18	GG	CC	65
Yannong19	AA	CC	65
Yanzhan4110	AA	CC	65
YangWheat	GG	CC	55
Yumai18	GG	CC	60
Yumai2	GG	CC	60
Yumai35	GG	CC	50
Yumai49	GG	CC	65
Yumai50	GG	CC	70
Yumai57	GG	CC	62
Yumai63	AG	CC	60
Changwu134	AA	CC	45
Zheng9023	GG	CC	60
Zhongmai875	GG	CC	60
Zhongmai895	GG	CC	60
Zhou8425B	AA	CC	58

Zhoumai11	AA	CC	20
Zhoumai22	GG	CC	55
Zhoumai23	GG	CC	70
Zhoumai30	GG	CC	60
Zhoumai31	GG	CC	55

Table S2. The candidate genes identified for leaf rust resistance

Candidate gene	Chr.	Physical position (Mb)	Annotation	Expressed at leaves
TraesCS1B01G277100	1B	484.7	Phospholipase A1	Yes
TraesCS1B01G277300	1B	484.9	NAC domain protein,	Yes
TraesCS1B01G277900	1B	486.5	Disease resistance protein (NBS-LRR class) family	Yes
TraesCS1B01G279300	1B	487.7	C2 calcium/lipid-binding and GRAM domain protein	Yes
TraesCS1B01G281100	1B	489.2	Kinase family protein	Yes
TraesCS1B01G281500	1B	489.7	Ethylene-responsive transcription factor RAP2-11	Yes
TraesCS1B01G459300	1B	673.2	Protein kinase family protein	No
TraesCS1B01G459500	1B	673.6	Indole-3-acetic acid-amido synthetase GH3.3	Yes
TraesCS1B01G463500	1B	675.6	F-box domain containing protein	No
TraesCS1B01G465900	1B	676.7	Leucine-rich repeat receptor-like protein kinase family protein	Yes
TraesCS1B01G466200	1B	676.7	Pathogen-related protein	Yes
TraesCS1B01G466300	1B	676.8	Phosphoglucan phosphatase LSF1	Yes
TraesCS1B01G466700	1B	676.9	Pm3-like disease resistance protein	No
TraesCS1B01G466900	1B	677.0	Disease resistance protein	Yes
TraesCS1B01G467000	1B	677.0	Leucine-rich repeat receptor-like protein kinase	Yes
TraesCS1B01G467700	1B	677.3	Lectin receptor kinase	Yes
TraesCS1B01G467800	1B	677.5	Receptor kinase-like protein	Yes
TraesCS1B01G469200	1B	678.3	Cytoplasmic membrane protein	Yes
TraesCS1B01G471300	1B	679.6	Cytokinin riboside 5'-monophosphate phosphoribohydrolase	Yes
TraesCS2A01G576700	2A	770.9	Serine/threonine protein phosphatase 7 long form isogeny	No
TraesCS2A01G578900	2A	771.9	receptor kinase 1	Yes
TraesCS2A01G580400	2A	772.7	Disease resistance protein RPM1	Yes
TraesCS2A01G580700	2A	773.0	F-box domain containing protein, expressed	No
TraesCS2A01G582100	2A	774.6	Glutathione peroxidase	Yes
TraesCS2A01G582400	2A	774.8	Receptor-like protein kinase	Yes
TraesCS2A01G584800	2A	776.0	Endo-1,4-beta-xylanase	No
TraesCS2A01G585200	2A	776.2	Disease resistance protein (TIR-NBS-LRR class) family	No
TraesCS2A01G586400	2A	777.1	E3 ubiquitin-protein ligase	Yes
TraesCS2A01G589500	2A	778.2	Sugar transporter, putative	Yes
TraesCS2A01G590400	2A	778.6	Sugar transporter, putative	No
TraesCS2A01G590500	2A	778.6	CAI-1 autoinducer sensor kinase/phosphatase cqsS isoform 1	No
TraesCS2A01G592100	2A	779.3	Lipoxygenase homology domain-containing protein 1	No
TraesCS2A01G592900	2A	779.8	Auxin-responsive protein	No
TraesCS2A01G593600	2A	780.7	Leucine-rich repeat receptor-like protein kinase family protein	No

TraesCS7B01G405400	7B	674.0	Ubiquitin carboxyl-terminal hydrolase family protein	Yes
TraesCS7B01G412300	7B	680.2	Zinc finger protein	Yes
TraesCS7B01G413700	7B	681.8	Phospholipase D Z	No
TraesCS7B01G413800	7B	681.8	Ubiquitin, putative	Yes
TraesCS7B01G433200	7B	700.7	Calcium-binding protein	No
TraesCS7B01G433700	7B	701.2	S-acyltransferase	No
TraesCS7B01G434700	7B	701.9	NF-X1-type zinc finger protein NFXL1	Yes
TraesCS7B01G436400	7B	702.5	Calcium-dependent lipid-binding domain protein	No
TraesCS7B01G436900	7B	703.2	TNF receptor-associated factor family protein	Yes
TraesCS7B01G437100	7B	703.3	Transcriptional corepressor SEUSS	Yes
TraesCS7B01G440000	7B	705.3	Protein kinase, putative	Yes
TraesCS7B01G440100	7B	705.7	Endo-1,31,4-beta-D-glucanase	No
TraesCS7B01G440400	7B	706.0	AP2-like ethylene-responsive transcription factor	Yes
TraesCS7B01G441200	7B	706.4	Receptor protein kinase-like protein	No
TraesCS7B01G441900	7B	706.8	Calcium-dependent lipid-binding domain-containing protein	Yes
TraesCS7B01G442800	7B	707.7	Ankyrin repeat family protein	No
TraesCS7B01G443500	7B	708.1	Zinc finger CCCH domain protein, putative	Yes
TraesCS7B01G443800	7B	708.5	MYB transcription factor	Yes
TraesCS7B01G446200	7B	709.5	Pathogenesis-related thaumatin family protein	Yes
